# Treadmill-based assessment of running-specific prostheses in steady and accelerated running: a case study with a Paralympic medalist

**DOI:** 10.3389/fspor.2026.1817303

**Published:** 2026-06-10

**Authors:** Samira G. Breban, Giuseppe Marcolin, Roberto Di Marco, Andrea G. Cutti, Gian Luca Migliore, Nicola Petrone

**Affiliations:** 1 Department of Industrial Engineering, University of Padova, Padova, Italy; 2 Department of Biomedical Sciences, University of Padova, Padova, Italy; 3 Centro Protesi, INAIL, Bologna, Italy

**Keywords:** Paralympic sprinters, running mechanics, running prosthetic foot, sprinting acceleration, treadmill

## Abstract

Running-specific prostheses (RSPs) enable individuals with lower-limb amputation to compete in Paralympic running disciplines. In a high-level sports context, optimal RSP tuning based on biomechanical parameters is essential to enhance running performance. Although on-track assessment is considered the gold standard, its high financial and logistical demands make treadmill-based setups valuable alternatives. Therefore, this single-case study aimed to present a method for assessing steady-state running (SSR) and resisted acceleration running (RAR) in an amputee athlete, comparing different RSP alignments. The experimental setup combined a commercial treadmill mounted over four dynamometric platforms with an eight-camera motion capture system, enabling the kinetic, kinematic, and spatiotemporal assessment of different RSP configurations. Differences in biomechanical parameters were observed between RSP configurations during both SSR and RAR, including variations in GRFs and corresponding impulses, as well as hip, knee, and ankle kinematics. However, the sensitivity of the proposed method in detecting biomechanical differences should be interpreted with caution, as it is based on a single Paralympic athlete. In conclusion, these findings suggest that the treadmill-based protocol may serve as a functional and controlled screening tool for preliminary performance-oriented RSP tuning, enabling the identification of promising configurations prior to definitive assessments on an instrumented athletics track.

## Introduction

Running-specific prostheses (RSPs) enable athletes with lower limb amputations to compete in sport activities, including running competitions. Among these, the 100-m sprint remains a hallmark of the Paralympic Games, and winners are considered “the world's fastest athletes” (1).

The 100-m sprint can be divided into three phases: the acceleration phase (0–30 m), the maximum steady-speed phase (30–90 m), and the deceleration phase (90–100 m) (2). This classification is used for both able-bodied athletes and athletes with lower-limb amputation. Each of these phases requires specific training methods to improve overall sprinting performance, as the acceleration and maximum speed are associated with distinct biomechanical outputs (3).

Moreover, sprint performance is influenced by multiple factors, such as equipment, environment, and psycho-physiological conditions (4). RSP tuning represents a valid intervention contributing to enhancing spatiotemporal parameters (5), and running speed (6). Tuning may involve variations in shape (7) and stiffness (8, 9) of the running prosthetic foot (RPF), as well as socket alignment in the sagittal plane relative to the RPF (10, 11).

Quantitative biomechanical parameters, particularly kinetic [e.g., ground reaction forces (GRFs)] and kinematic (e.g., joint angles) parameters, are essential for evaluating the effects of RSP tuning on sprint performance. Indeed, as reported by Migliore et al. (10), specific alignments compared with others may reduce prosthetic-side hip extension at foot-off (FO) while preserving hip range of motion (ROM), decrease hip moment impulse, and enhance horizontal propulsion, thereby improving both comfort and performance. Ideally, such assessments should be conducted on track using instrumentation that does not hinder or restrict the athlete's natural running technique, thus allowing realistic competitive conditions to be reproduced. However, biomechanical analysis of a 100-m sprint in such settings is resource-intensive; for example, Nagahara and colleagues used 54 embedded force platforms (12). Although setups based on dense arrays of force platforms can provide high-quality data, their substantial logistical and financial demands may limit their practical application. To address these constraints, instrumented treadmills have become an increasingly accessible and widely adopted alternative for biomechanical assessment (13–17). Indeed, treadmill running equipped with force platforms provides a controlled testing environment, as the imposed belt speed allows the collection of consistent and repeatable consecutive steps compared with overground running (18). Moreover, treadmills are widely accepted in biomechanical running analysis (19) and have been used to predict in-field performance (18). However, instrumented motorized treadmills hardly reproduce the sprinting acceleration phase, which is crucial for achieving high running velocities (2). Consequently, they have been primarily used for assessment at steady-state speeds (10, 13, 20). In contrast, passive treadmills may represent a more suitable alternative for investigating sprint acceleration, as they allow the athlete to directly generate belt motion through force application and can be combined with external resistance to better simulate horizontal inertial demand. However, such systems specifically designed to evaluate sprint acceleration remain scarce (21) and have been used only with able-bodied athletes (22, 23). To our knowledge, no studies have investigated the sprint acceleration phase on treadmills in athletes with lower-limb amputations.

In light of the findings of Migliore et al. (10), which highlighted the importance of prosthetic alignment in influencing running performance, this single-case study aimed to compare two RSP tuning configurations (ST9° and ST15°), representing a more closed and a more open alignment, respectively. Using a controlled and repeatable treadmill-based setup with four force platforms, we investigated how these two alignment conditions affect kinetic and kinematic parameters. We hypothesized that the more open alignment (ST15°) would result in greater anterior–posterior (AP) GRF propulsive peaks and corresponding impulses. In addition, we expected that this configuration would be associated with a more preflexed hip angle compared with the more closed alignment (ST9°).

## Materials and methods

### Participants

One female athlete with left transfemoral amputation (mass: 55 kg, height: 1.60 m, age: 19, classification: T63), and a 100-m medalist in the 2020 Paralympic Games, participated in this study. The data collection protocol was implemented as part of her standard assessment routine and training during the 2023–2024 Paralympic season to support certified prosthetists in evaluating different RSPs for elite sprinters in preparation for the Paralympic Games in Paris. Written consent to disclose the data was obtained prior to testing from the athlete. During tests, she wore a 1E91 Standard Runner Cat 3.5 RPF (Ottobock, Germany) and a 3S80 monoaxial prosthetic knee joint (Ottobock, Germany).

### Experimental protocol

The experimental protocol was designed to assess biomechanical parameters associated with different RSP tuning configurations under controlled and repeatable conditions. In particular, running tests were performed on a commercial treadmill (SkillRun, Technogym, Italy) at the Department of Industrial Engineering, University of Padova. The treadmill was positioned over four force platforms (P-6000, BTS, Italy) operating at 1,000 Hz, with each of its four support feet centered on one separate platform to allow accurate measurement of GRFs.

**Figure 1:**
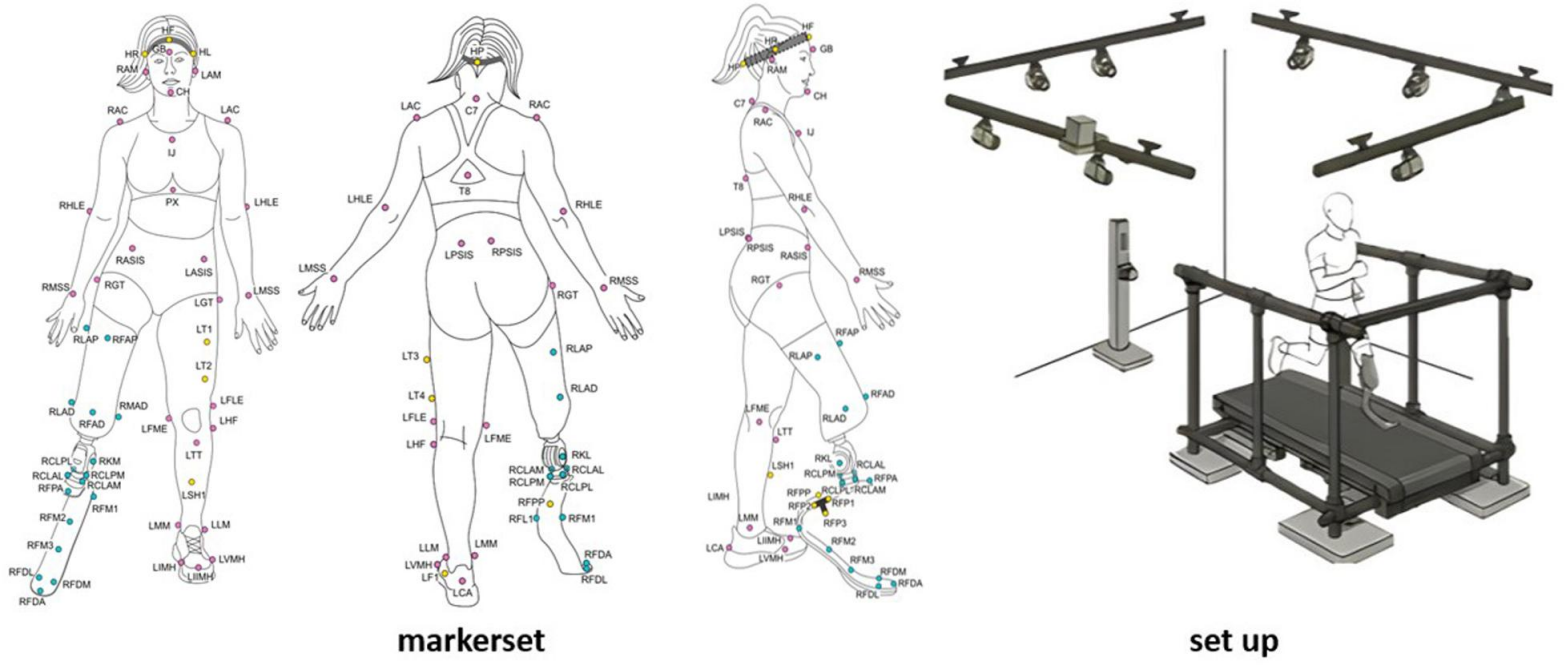
On the left, the marker set adopted in the study (24); on the right, the schematic representation of the experimental setup.

The setup (Figure 1) was located within the calibrated volume of an eight-camera motion capture system (SMART DX-6000, BTS, Italy), operating at 250 Hz. The cameras were arranged in a squared configuration around the treadmill, while the handrails and monitor of the treadmill were replaced with an external support frame to minimize the occlusion of markers. A safety harness was used to secure the athlete to the ceiling in case of a fall during the sprints.

Retro-reflective markers were placed on anatomical landmarks of the athlete's body and prosthesis (Figure 1), as described elsewhere (24). The athlete then performed a familiarization phase, which also served as a warm-up, consisting of walking and running at self-selected speeds for 15 min.

Two running conditions were considered: steady-state running (SSR) and resisted acceleration running (RAR). For each condition, trials were performed with the socket tilt set to 9° (ST9°) and 15° (ST15°) relative to gravity in the sagittal plane (10). For both socket alignments, the anterior displacement of the tip of the unloaded RPF relative to the line connecting hip and knee joint centers was set to 55 mm with the knee fully extended. The ST9° and ST15° alignment configurations are commonly adopted by athletes based on the clinical experience of prosthetic technicians, with ST15° representing the standard prosthetic alignment. Although the absolute difference between the two tested alignments is small (6°), it is relatively large compared with the ranges reported in previous studies (10).

During SSR, the treadmill operated in active mode, with speed gradually increasing from zero to 5 m/s over 40 s, corresponding to approximately 70% of the athlete's estimated maximal speed based on her 100-m best race time. Once 5 m/s was reached, the athlete maintained this speed for 12 consecutive steps.

During RAR, the treadmill operated in passive mode and a preloading elastic band was used to simulate inertial horizontal force. One end of the elastic band was attached to the athlete's harness at pelvis height, while the other end was connected to a uniaxial load cell (TF022, CCT, Italy; full scale: 1,000 N; acquisition frequency: 1,000 Hz) positioned at the rear of the treadmill. The load cell was statically preloaded at 110 N with the athlete standing in the middle of the treadmill belt. This force corresponded to a resistance of approximately 20% of body weight (BW), a value previously adopted for sprint training and assessment (23). The athlete was instructed to perform a maximal sprint, accelerating until reaching her peak velocity, which was then maintained for 5 s. The first 20 consecutive steps were retained for further analysis.

Four repetitions were performed for each alignment in the following order: three SSR trials followed by one RAR trial. A 3-min rest interval was provided between trials. Recovery periods were consistent with those typically adopted by the athlete during training with similar exercises to minimize fatigue. Although this was a single-case study, the ST9° and ST15° alignments were randomized.

### Data analysis

For the SSR condition, the 12 consecutive steps were divided into six steps of the affected limb (AL) and six steps of the unaffected limb (UL). Within each trial, steps were averaged separately for AL and UL. Subsequently, the mean values obtained from the three trials were averaged to yield a representative value for each limb.

For the RAR condition, starting from the first ground contact of AL, three steps per limb were selected to represent the initial (INIT: 1st AL and 2nd UL), middle (MID: 11th AL and 12th UL), and final (FIN: 19th AL and 20th UL) phases of acceleration.

#### Kinematic parameters

Running speed was determined differently for the two conditions. In RAR, it was calculated as the time derivative of the anterior–posterior trajectory of a marker placed on the treadmill belt, with the resulting values fitted using a seventh-order polynomial. In SSR, speed was directly set by the treadmill. Markers trajectories were filtered using a fourth-order zero-lag low-pass Butterworth filter (10 Hz cutoff frequency). Local coordinate systems and joint kinematics followed previously described procedures (24). Marker positions were optimized to accommodate skin artifacts and instrumental errors using singular value decomposition (25). Joint kinematics were computed from the relative rotations between two adjacent segments, using appropriate Cardan sequences: zx′y″ for hip and knee angles, and zy′x″ for the unaffected ankle joint. Kinematic parameters were calculated for the hip, knee, and ankle joints of both the AL and UL limbs following the procedures described elsewhere (24). Only sagittal plane kinematics (flexion–extension angles) were considered. Joint angle trajectories were time-normalized over the stride cycle, from which discrete variables were extracted, including peak values, ROM, and values of joint angles at foot-off.

#### Kinetic parameters

GRFs were obtained in the ground reference system of the motion capture setup, with *X*-anterior–posterior axis pointing forward, *Y*-vertical (V) axis pointing upward, and *Z*-medial–lateral axis pointing rightward. As the force platforms were properly calibrated and synchronized, the resultant GRF was obtained by summing the signals from the four force platforms, resulting in an estimated uncertainty of approximately 2% of full scale (8,000 N). Notably, because only one limb is in contact with the ground during running, the GRFs collected could be clearly attributed to each limb. After spectral analysis, a fourth-order, zero-lag Butterworth low-pass filter with a 15-Hz cutoff frequency was applied to the force signals, which preserved 86% of the signal power. Foot contact (FC) and FO were detected from the filtered vertical GRF signal by applying a threshold of 100 N.

During the stance phase of each limb, braking (IHb) and propulsive (IHp) impulses were calculated as the time integrals of the negative and positive values of the GRF_AP_, respectively. The net AP impulse (IHn) was obtained as the sum of IHb and IHp for each step. Similarly, the vertical impulse (IV) was calculated as the area under the GRF_V_ curve during stance for both AL and UL. All GRFs and impulses were normalized to the subject's BW, including the weight of the running prosthesis.

Vertical average loading rate (VALR) was calculated as the ratio between the difference in GRF_V_ at 20% and 80% of the time to peak impact and the time elapsed between these two events: $\left(GRF_{V80\text{\%}}-\;GRF_{V20\text{\%}})/(t_{80\text{\%}}\;-\;t_{20\text{\%}}\right)$ (17).

## Results

Results for SSR are presented as mean and standard deviation of the six strides analyzed for AL and UL, separately. Conversely, for RAR, results of INIT, MID, and FIN steps are presented as single values. In RAR, the maximum speed achieved was 3 m/s for both alignments.

### Kinetics results

**Figure 2:**
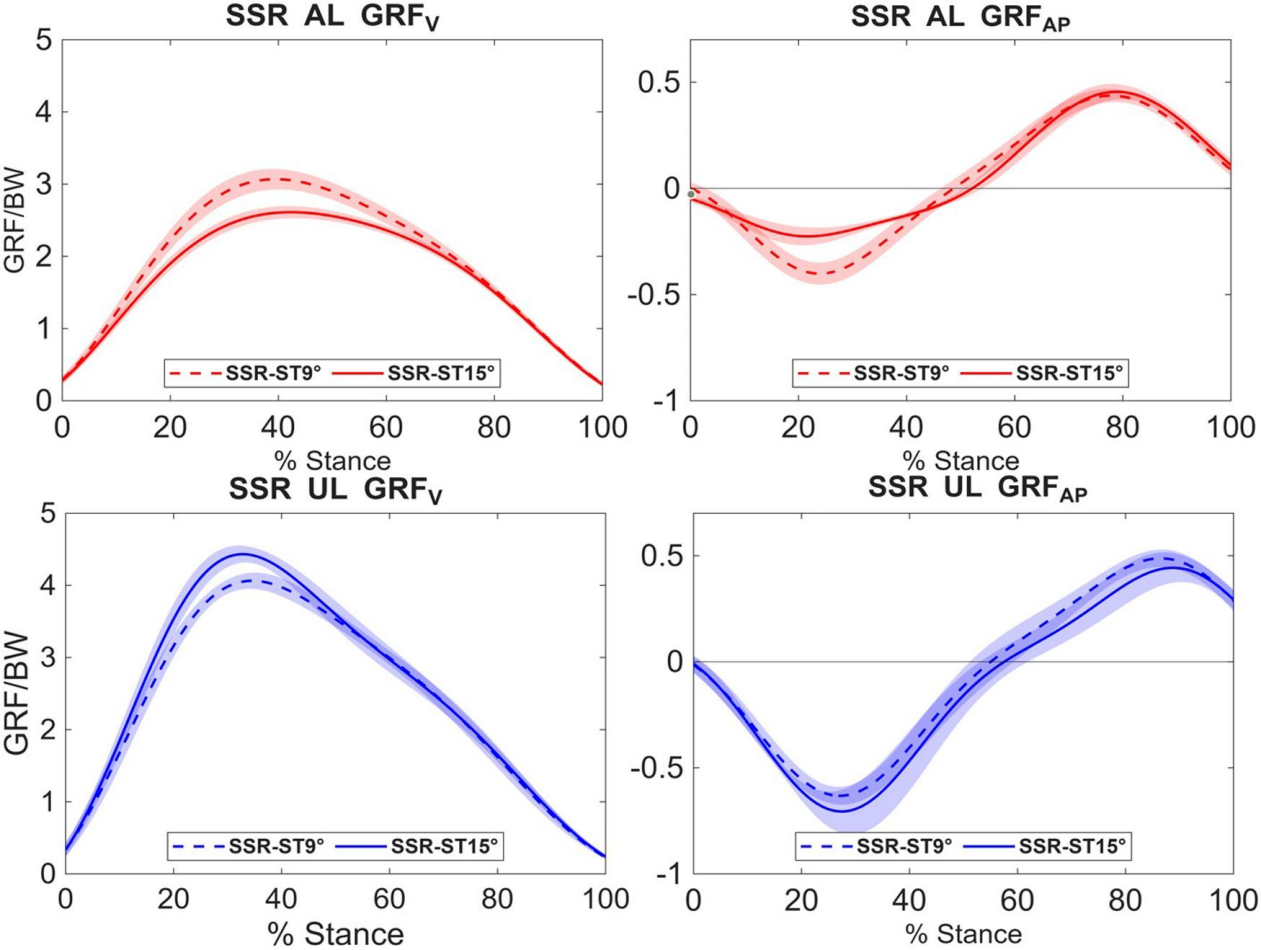
SSR GRFs of AL (top) and UL (bottom) are shown for ST9° and ST15° alignments. For each GRF component, the mean curve and standard deviation are reported. Solid lines refer to the ST15°, whereas dash-dot lines refer to ST9°.

In SSR, the GRF_V_ peak was 9% higher in ST15° (4.43 BW) than ST9° (4.06 BW) for UL, but 14% lower in AL (2.61 BW) compared with UL (3.07 BW). As for GRF_AP,_ during SSR, UL exhibited a breaking GRF_AP_ (−0.63 BW with ST9° and −0.70 BW with ST15°) greater than AL (−0.40 BW with ST9° and −0.23 BW with ST15°). Conversely, the propulsive GRF_AP_ values were similar for both alignments (UL: 0.48 BW with ST9° and 0.44 BW with ST15°; AL: 0.44 BW with ST9° and 0.45 with ST15°). Figure 2 illustrates the described results.

**Figure 3:**
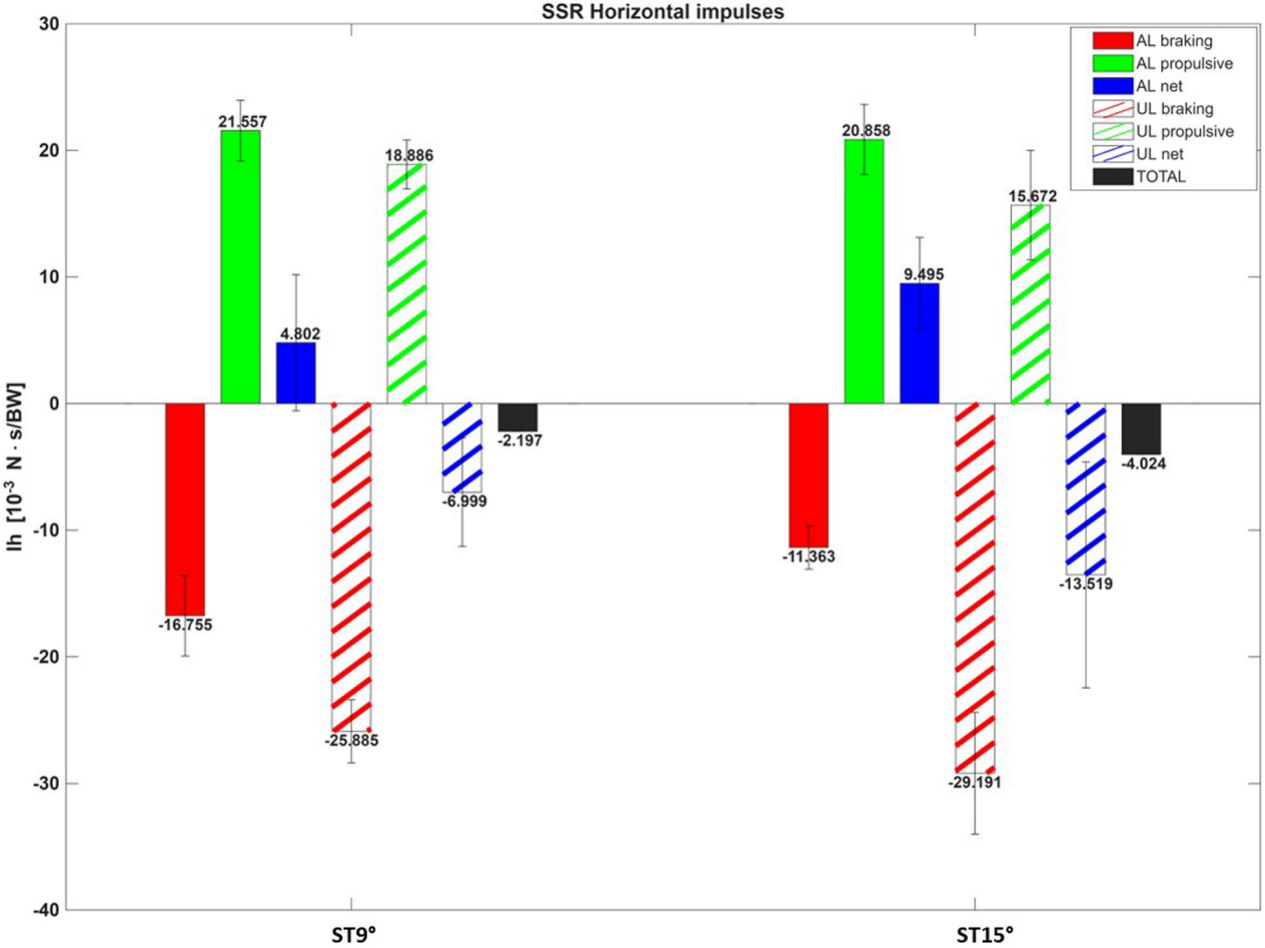
SSR horizontal impulses for ST9° and ST15° alignments. For each limb, IHb, IHp, and IHn impulses are presented.

Regarding SSR impulses (Figure 3), UL showed greater IHb than AL, with higher values in ST15° compared with ST9°. Moreover, UL generated smaller IHp than AL with both ST9° (−12%) and ST15° (−25%). Consequently, IHn assumed negative values for UL and positive values for AL. Impulse values shown in the figure were scaled by a factor of 10^3^ to improve readability, given the very small magnitude of the original measurements.

**Figure 4:**
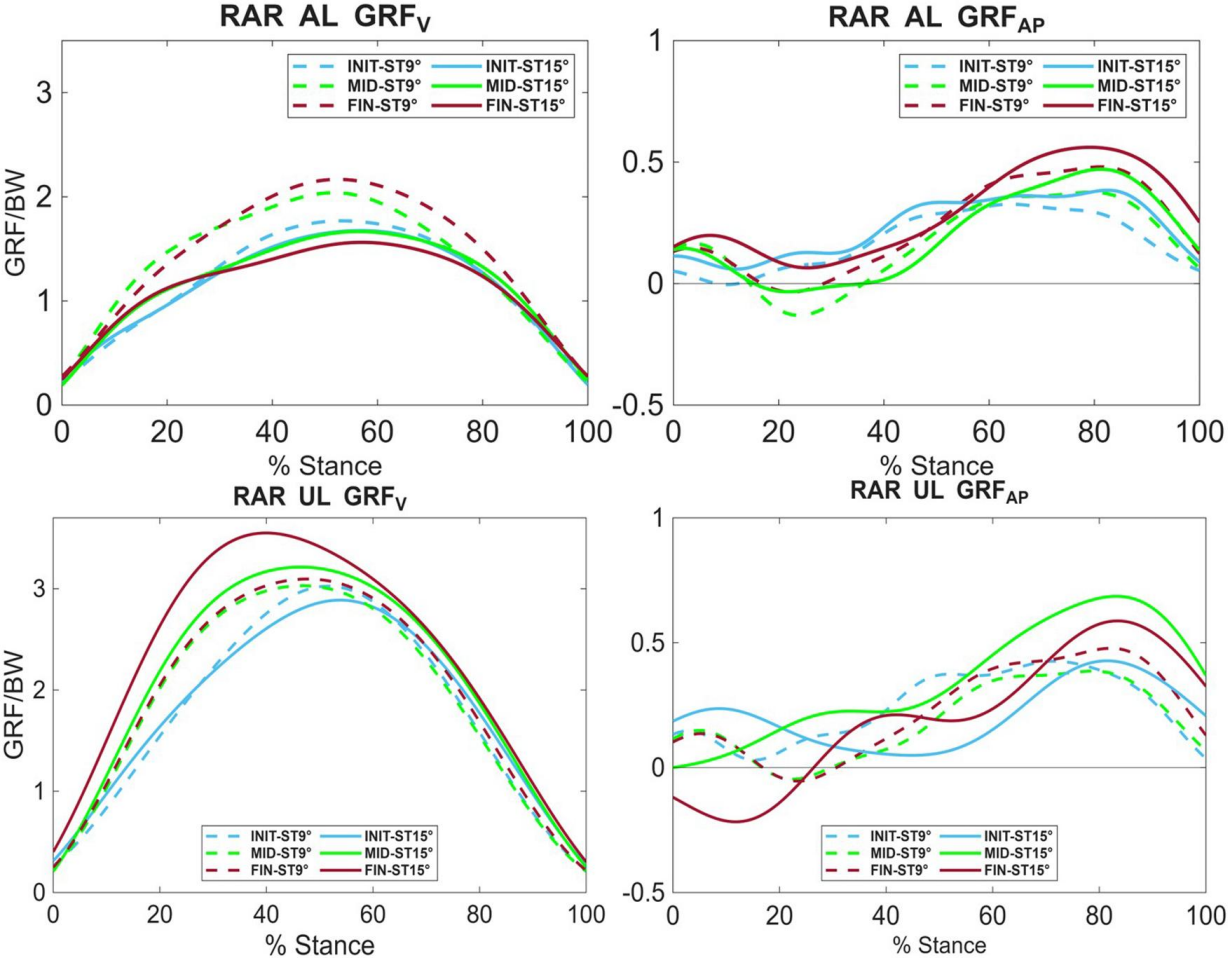
RAR GRFs of AL (top) and UL (bottom) are shown for ST9° and ST15° alignments. For each GRF component, the curves of the INIT, MID, and FIN steps are presented. Solid lines refer to the ST15°, whereas dash-dot lines refer to ST9°.

In RAR, FIN GRF_V_ peaks with ST9° were 2.16 BW and 3.10 BW for AL and UL, respectively, whereas with ST15° they were 1.56 BW for AL and 3.54 BW for UL. As for GRF_AP_, propulsive GRF_AP_ values were 0.47 BW and 0.50 BW, respectively, for AL and UL with ST9°. In ST15°, propulsive peak GRF_AP_ values were 0.56 BW for AL and 0.69 BW for UL. Figure 4 presents the RAR GRFs for both AL and UL.

**Figure 5:**
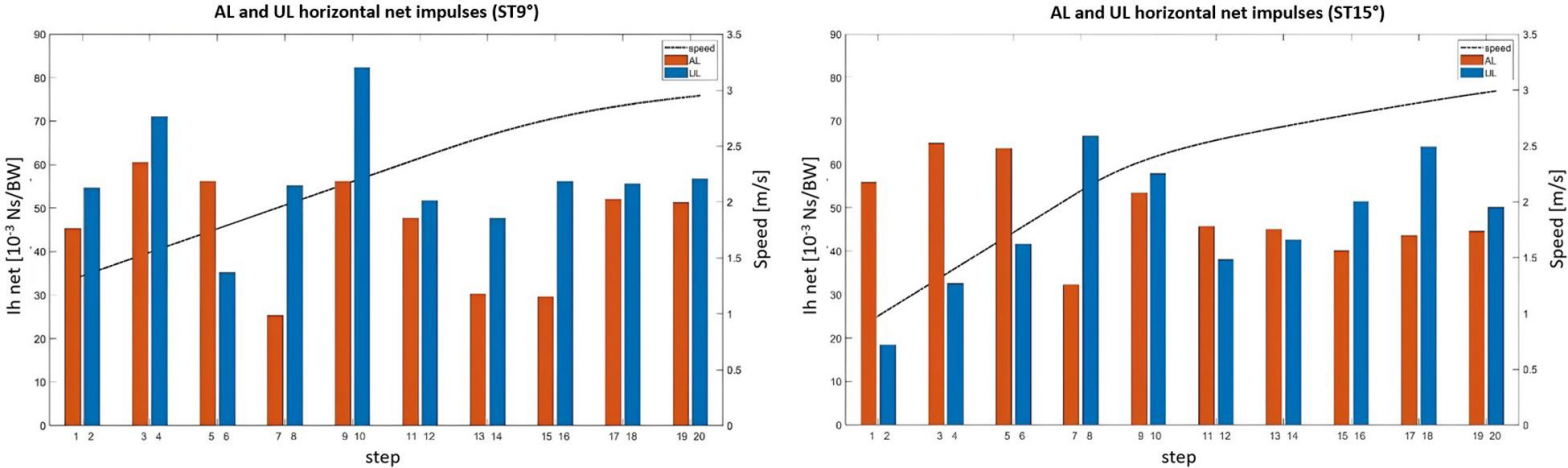
RAR IHn for both limbs with ST9° (left) and ST15° (right) alignments with respect to step number and instantaneous speed.

Figure 5 presents the RAR horizontal impulses of the first 20 steps, together with the instantaneous speed of the athlete. In addition to the impulses of the three selected steps—representing the initial (INIT: 1st AL and 2nd UL), middle (MID: 11th AL and 12th UL), and final (FIN: 19th AL and 20th UL) phases of acceleration—impulses for the remaining 14 steps are also shown. This allows a comprehensive overview of the progression of impulses throughout the acceleration phase. Unlike SSR, IHn was positive for both AL and UL. In ST9°, except for steps 5–6, IHn of AL was generally lower than IHn of UL. In ST15°, IHn of AL was greater than IHn of UL for all steps, with the exception of steps 7–8 and 15–20. Impulse values shown in the figure have been scaled by a factor of 10^3^ to improve readability, given the very small magnitude of the original measurements.

In SSR, AL exhibited longer contact times than UL, with increases of 15% and 13% in ST9° and ST15°, respectively. UL VALR values were 40% and 57% higher than those of AL in ST9° and ST15°, respectively. Table 1 presents the described results.

**Table 1:** Impulses and spatiotemporal parameters in SSR tests.

Alignment	Side	Vertical impulse (Ns/BW)		Stance time (s)		VALR (BW/s)	
		Mean	SD	Mean	SD	Mean	SD
ST9°	AL	0.152	0.012	0.161	0.004	52.385	4.316
	UL	0.195	0.008	0.139	0.003	86.955	3.611
ST15°	AL	0.116	0.010	0.158	0.002	42.019	1.815
	UL	0.211	0.006	0.140	0.004	97.922	5.653

During RAR, contact time progressively decreased with increasing speed for both limbs; however, as expected, the minimum contact time observed (steps 19–20) remained higher than the corresponding SSR values. In RAR, VALR increased with speed and was consistently greater for UL across both alignments. Table 2 presents the RAR results for the INIT, MID, and FIN steps for both limbs.

**Table 2:** Impulses and spatiotemporal parameters in RAR tests.

Alignment	Side	Vertical impulse (Ns/BW)	Stance time (s)	VALR (BW/s)
ST9°	AL step INIT	0.058	0.256	13.032
	AL step MID	0.089	0.240	16.472
	AL step FIN	0.097	0.212	19.889
	UL step INIT	0.380	0.216	32.590
	UL step MID	0.339	0.192	42.627
	UL step FIN	0.351	0.164	52.142
ST15°	AL step INIT	0.049	0.232	11.512
	AL step MID	0.047	0.212	11.712
	AL step FIN	0.032	0.188	13.445
	UL step INIT	0.303	0.160	35.259
	UL step MID	0.346	0.156	47.084
	UL step FIN	0.334	0.148	63.555

### Kinematics results

**Figure 6:**
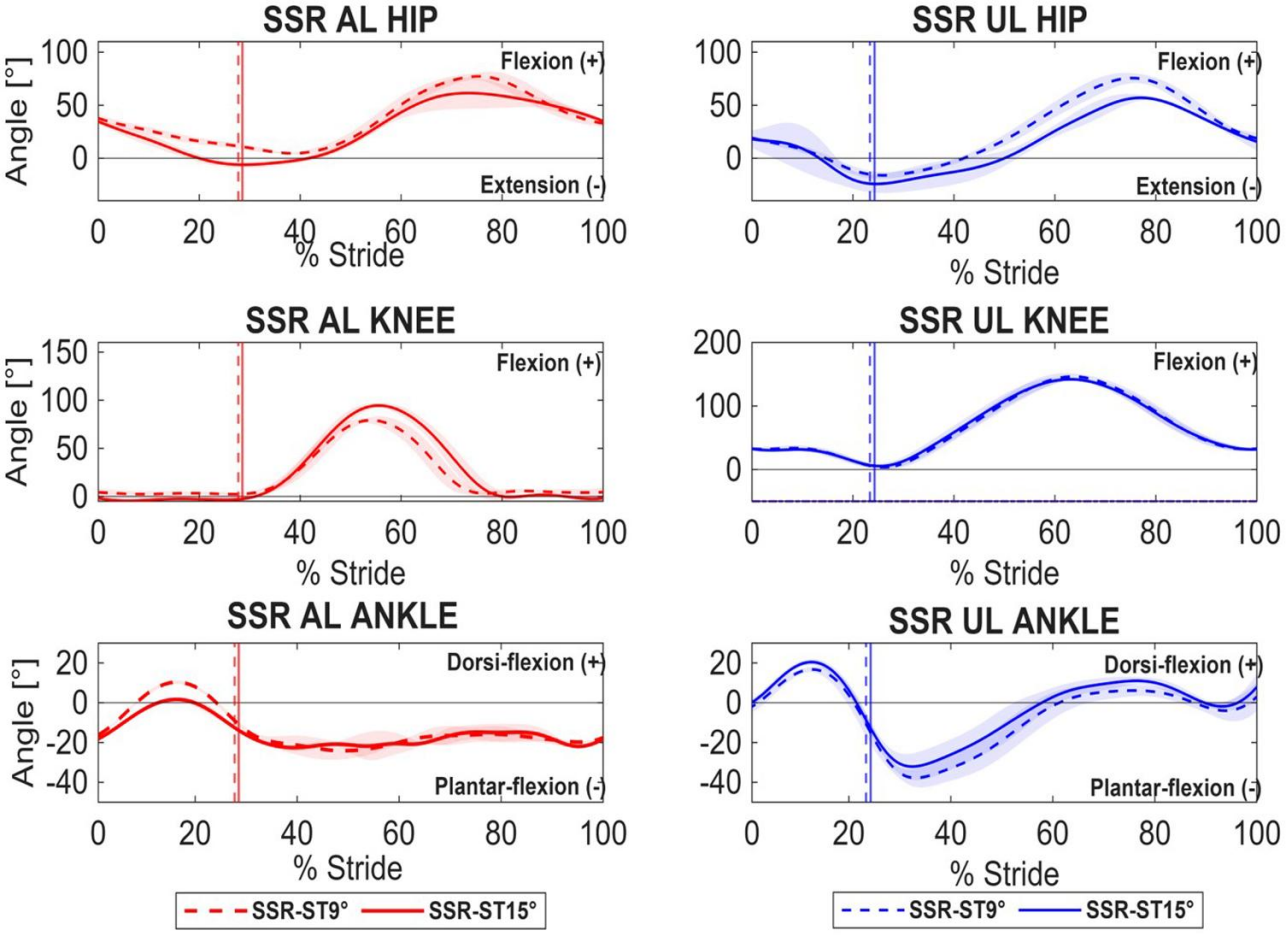
SSR sagittal joint angles for both AL and UL hip, knee, and ankle are shown. For each alignment, the mean curve and standard deviation obtained during the SSR test are presented. Solid lines refer to the ST15° alignment, whereas dashed lines represent the ST9° alignment. The vertical lines indicate FO.

Figure 6 shows AL and UL changes in sagittal joint angles for ST9° and ST15°, respectively, during SSR tests. Maximum UL hip extension occurred at FO, with values of −15° (ST9°) and −24° (ST15°). Conversely, the maximum UL hip flexion was reached within the swing phase with values of 75° (ST9°) and 60° (ST15°).

In SSR, focusing on the AL hip at FO, values were 12° with ST9° and −4° with ST15°, whereas in the FIN phrase they were 8° and 7° for ST9° and ST15°, respectively. During the SSR swing phase, the maximum AL hip flexion was 76° with ST9° and 59° with ST15°. The UL knee joint exhibited two physiological flexion patterns: a small flexion during the stance phase and a larger flexion during the swing phase. Conversely, the AL knee, functioning as a locked mechanical hinge, maintained a constant angle throughout the stance phase and flexed only during the swing phase, regardless of whether SSR or RAR was considered. In SSR, UL knee flexion was on average 32° at FC and 7° at FO. Moreover, in SSR, AL knee peak flexion was 79° with ST9° and 94° with ST15°. With regard to the ankle joint, in SSR, UL peak dorsiflexion was 13° in stance and 6° in swing with ST9°, and 20° in stance and 10° in swing with ST15°. Plantarflexion was greater in ST15° (−38°) than ST9° (−32°). AL exhibited a dorsiflexion peak of 9° with ST15° and 2° with ST9°.

**Figure 7:**
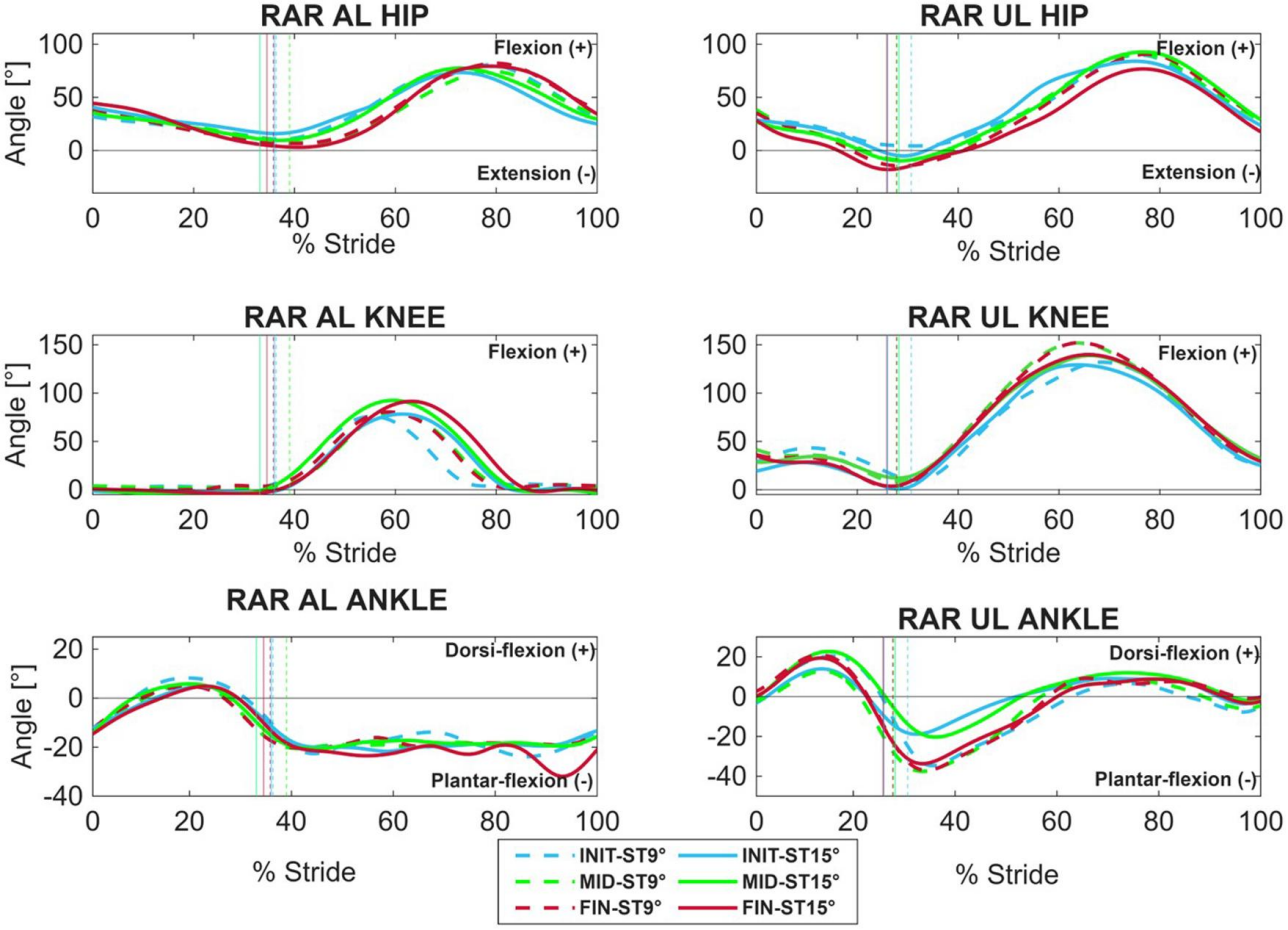
RAR sagittal joint angles for both AL and UL hip, knee, and ankle are shown. For each alignment, the curves of the INIT, MID, and FIN steps are presented. Solid lines refer to the ST15° alignment, whereas dashed lines represent the ST9° alignment. The vertical lines indicate FO.

As for RAR (Figure 7), maximum UL hip extension occurred at FO, with values of −13° (ST9°) and −16° (ST15°). Conversely, maximum UL hip flexion was reached during the swing phase with values of 90° (ST9°) and 92° (ST15°) in the FA RAR. In RAR, UL knee angle at FC was higher than at FO, with different ranges depending on the alignment. In particular, with ST9°, angles at FC ranged from 28° to 40°, whereas with ST15° they ranged from 20° to 35°. During the swing phase, apart from INIT, UL knee reached similar peak flexion values in both SSR and RAR tests. In RAR, AL knee exhibited a flexion range of 70°–80° with ST9° and 78°–91° with ST15°.

Ankle joint peaks were similar in RAR, when comparing ST9° with ST15°.

## Discussion

The present study aimed to develop a method for analyzing the biomechanics of the early acceleration and flying phases of sprinting within a controlled and repeatable framework for performance-oriented prosthetic assessment. To achieve this goal, a commercial treadmill was mounted over four dynamometric platforms, and a motion capture system collected sagittal kinematics. Overall, the kinetic and kinematic results demonstrated that the proposed treadmill-based method was able to detect differences in prosthetic alignment configurations within each type of condition (i.e., SSR and RAR). These differences were reflected in GRF peaks and impulses, as well as in ROM and peak joint angles at the hip, knee, and ankle. In addition, the method proved effective in distinguishing between AL and UL conditions, consistent with findings in the existing literature (26).

We calculated impulses and VALR, which are considered the main biomechanical parameters when examining running biomechanics (27), together with joint kinematics and spatiotemporal parameters. Impulses have been reported as important contributors to forward velocity (13), while VALR is also considered an indicator of athlete injury risk (27). Joint kinematics provide information about running technique, and stance time has recently been identified as one of the determinants of running speed in Para-athletes (20).

Our findings aligned with previous studies (26, 28) regarding differences in GRFs between AL and UL. In particular, in our results, UL consistently exhibited greater vertical and horizontal peaks than the AL during both SSR and RAR, independent of socket alignment. With respect to running speed, vertical GRF in our RAR trials increased with acceleration, in line with previous studies (26, 29). Furthermore, horizontal impulses were mainly propulsive, as highlighted in prior work (30). Conversely, in SSR, we observed an initial braking phase followed by a propulsive horizontal impulse, consistent with previous studies (10, 13).

During SSR, the sagittal range of motion of UL joints was lower in ST15° than in ST9°, with the greatest reduction observed at the hip joint. This suggests that adjustments to accommodate the prosthesis may be more pronounced at this joint. In our interpretation, the kinematics observed with ST15° may contribute to greater interlimb symmetry and a more regular running pattern. Our findings on knee angles aligned with previous measurements on track (31, 32), showing that UL knee reached a higher peak flexion angle than AL knee during the swing phase. Moreover, the decrement of UL knee flexion with speed was in accord with previous studies (33, 34).

The shorter UL stance time compared with the AL in both SSR and RAR aligns with Grabowski et al. (35), who observed that force production differs between limbs. On the prosthetic side, lower force is compensated by increased contact time, which is influenced by the elastic response of the RPF and the athlete's mass. This interaction allows maintenance of a comparable propulsive impulse during sprinting. Regarding loading rate, the greater UL VALR compared with AL for both SSR and RAR is consistent with previous findings in unilateral transfemoral amputees (27). Interestingly, given that 70 BW/s has been proposed as an injury-related threshold (32), the values exceeding this limit in the UL may reflect potential exposure to higher mechanical loading, without implying a direct injury risk.

Unlike other studies in the literature that focus solely on steady-state running using instrumented treadmills, our method also includes the acceleration phase. Importantly, our steady-state treadmill results were consistent with previously reported literature values, suggesting that the proposed approach provides reliable measurements even during the acceleration phase.

Nonetheless, the present study has some limitations that should be acknowledged. First, data were collected from a single athlete; thus, the observed differences should be interpreted with caution, and the sensitivity and robustness of the method should be further investigated in larger cohorts of athletes. Second, although the observed kinematic outcomes were consistent with existing literature, the treadmill-based experimental setup did not allow athletes to reach typical overground sprinting speeds. This limitation likely resulted in lower peak forces and impulses compared with overground sprinting.

## Conclusions

Considering its strengths and limitations, the proposed treadmill-based method may represent a practical tool for orthopedists, coaches, and athletes to perform preliminary screening of running-specific prosthesis designs and tunings. In particular, the ability to analyze a large number of consecutive strides through combined kinematic and kinetic measurements may support a more informed selection of promising configurations, potentially reducing the number of RSP setups to be tested in subsequent, more demanding track-based assessments. However, caution is warranted given the single-case nature of this study. Further investigations involving larger cohorts of athletes are required to confirm the generalizability, reliability, and sensitivity of the proposed approach across different performance levels and prosthetic configurations.

## Data Availability

The original contributions presented in the study are included in the article/Supplementary Material, further inquiries can be directed to the corresponding author.
